# Relevance of sleep and associated structural changes in GBA1 mouse to human rapid eye movement behavior disorder

**DOI:** 10.1038/s41598-022-11516-x

**Published:** 2022-05-13

**Authors:** Cigdem Gelegen, Diana Cash, Katarina Ilic, Millie Sander, Eugene Kim, Camilla Simmons, Michel Bernanos, Joana Lama, Karen Randall, Jonathan T. Brown, Svjetlana Kalanj-Bognar, Samuel Cooke, K. Ray Chaudhuri, Clive Ballard, Paul Francis, Ivana Rosenzweig

**Affiliations:** 1 Department of Neuroimaging, Sleep and Brain Plasticity Centre, Institute of Psychiatry, Psychology and Neuroscience (IoPPN), King’s College London (KCL), De Crespigny Park, Box 089, London, SE5 8AF UK; 2 Basic and Clinical Neuroscience, IoPPN, KCL, London, UK; 3 BRAIN, Department of Neuroimaging, KCL, London, UK; 4 Croatian Institute for Brain Research, University of Zagreb School of Medicine, Zagreb, Croatia; 5 College of Medicine and Health, University of Exeter, Exeter, UK; 6 Institute of Psychiatry, Psychology and Neuroscience, Wolfson Centre for Age-Related Diseases, Guy’s Campus, KCL, London, UK; 7 King’s College London and Parkinson’s Foundation Centre of Excellence, King’s College Hospital, London, UK; 8 Sleep Disorders Centre, GSTT, London, UK

**Keywords:** Neuroscience, Medical research, Neurology

## Abstract

Rapid eye movement (REM) sleep behaviour disorder (RBD) is a REM parasomnia that often predicts the later occurrence of alpha-synucleinopathies. Variants in the gene encoding for the lysosomal enzyme glucocerebrosidase, GBA, strongly increase the risk of RBD. In a GBA1-mouse model recently shown to mimic prodromal stages of α-synucleinopathy, we now demonstrate striking REM and NREM electroencephalographic sleep abnormalities accompanied by distinct structural changes in the more widespread sleep neurocircuitry.

## Introduction

Idiopathic (isolated) rapid eye movement (REM) sleep behaviour disorder (iRBD) is a type of REM parasomnia^1–3^, which represents the prodromal stage of an α-synucleinopathy in more than 80% of patients^3–5^. Variants in the gene encoding for the lysosomal enzyme glucocerebrosidase, GBA, strongly increase the risk of iRBD^6,7^. The rate of conversion to neurodegeneration is also increased, and may be faster among severe GBA variant carriers^7^. This has led some clinicians to argue for more regular screening for iRBD in all healthy carriers of GBA variants in order to identify carriers who will develop α-synucleinopathy in the future^6^.

Elementary, minor and major body and limb jerks on surface electromyography (REM sleep without atonia) or video polysomnography are a hallmark of iRBD^3,8^. However, to date, surprisingly little is still known about the (microscopic) sleep architecture in iRBD^9–12^. Even less is known about its relationship with the GBA-genotype and any associated neuroanatomical or circuitry changes^13^. In the past, it has been argued that a breakdown in the specific “REM-on” brainstem sleep circuitry residing in the region of the sublaterodorsal tegmental nucleus and the coeruleus/subcoeruleus locus (SLD)^14^, i.e. the structure analogous to the peri-locus coeruleus-alpha in cat and sublaterodorsalis nucleus (e.g. A6sc) in rodents, might lead to iRBD-associated sleep architectural changes^9,15–17^. However, the specific changes have by and large remained elusive^17^. In order to put this concept to the test, whilst accounting for the effect of GBA1-genotype, we used a GBA1-mouse model (heterozygous D409V/WT), recently shown to mimic early prodromal stage of α-synucleinopathy^18^.

## Methods and materials

Three mouse lines were investigated, heterozygous (D409V/WT), homozygous (D409V/D409V) GBA1 mutant mice (D409V/WT) and wild type (C57Bl/6), (further in the text D409V/WT, D409V/D409V and WT respectively), all previously described by us and others^18,19^ (Supplement Methods; Fig. S1 depicts the research protocol). D409V/WT (heterozygous GBA1 mice) carry one copy of the human D427V point mutation (human equivalent of the murine D409V point mutation) in the murine *glucocerebrosidase* (*Gba*) gene. Video-electroencephalogram (EEG)-recordings and polysomnography investigations were done according to a strict twelve-hour light–dark cycle protocol, as previously described^20^. All investigations were performed in accordance with the United Kingdom Home Office Animal Procedures Act (1986) and approved by the King’s College London Animal Welfare Ethical Review Body (AWERB).

### Sleep recording and EEG analysis

Ten heterozygous D409V/WT and eight WT mice were implanted with screw electrodes (10–50 kΩ) (Supplement, Fig. S2). The monopolar screw electrodes record the frontal and parietal EEG with ground at occipital bone. In addition, a pair of stainless-steel EMG electrodes was implanted in the dorsal neck muscle. The electrodes were secured with dental cement and standard EEG was recorded for twenty-four hours. EEG/EMG signals were sampled at 200 Hz, amplified 100 × and low-pass filtered at 100 Hz using a pre-amplifier (please refer to Supplement Methods for further in depth description). Sleep scoring was performed manually on ten-second epochs using *Sirenia Sleep* software (*Pinnacle Technology Inc*.), and signal processing analysis was conducted as previously described^21^. Further analysis of EEG data in the frequency domain was performed using Fourier transforms. Spectrograms showing the amplitude of EEG signals in the time and frequency domain were generated using either Morlet wavelet^22^ or using short-time Fourier transform with 1024 samples window length, as previously described^23^. EEG signal analyses were conducted using custom codes written in MATLAB, as previously described^23–25^.

All statistical tests were performed in “GraphPad Prism” or IBM SPSS Statistics version 25.0. Kolmogorov–Smirnov test was used for normality. Data are represented as the mean ± SEM, unless otherwise stated. Two-way ANOVA (time and treatment factors) and two-tailed unpaired *t-*test were used for the analysis of the sleep and EEG spectrum power data. *P* values are shown when they are less than 0.05 (*please refer to Supplement for further in-depth description and the full list of methodological references*).

### Neuroimaging methodology

MRI and Statistical Analyses: The MR images were acquired on 9.4 T scanner from ex vivo brains and processed using a combination of FSL, ANTs and the QUIT toolbox, as previously described by our group^26^. Tensor based morphometry (TBM) was used to assess the effect of genotype on regional brain volumes. A group comparison was carried out on the Jacobian determinant images with permutation tests and Threshold-Free Cluster Enhancement (TFCE) using FSL randomise. Voxelwise differences data (Supplement, Figs. S4–S6) are displayed on the mouse template image. We also performed a region of interest (ROI) analysis using 71 anatomical ROIs derived from a modified Allen brain atlas, that we co-registered to the study template used for TBM. Volumes of each ROI were calculated by summing the Jacobian determinants within the ROI, and univariate pairwise group comparisons performed using Mann–Whitney U-tests. (please refer to Supplement for the full list of methodological references).

Further detailed methodological description of experimental and study procedures, including the undertaken statistical and MR analyses and all the pertinent methodological references, are available in the Supplement. The study is reported in accordance with ARRIVE guidelines^27^.

## Results

**Figure 1:**
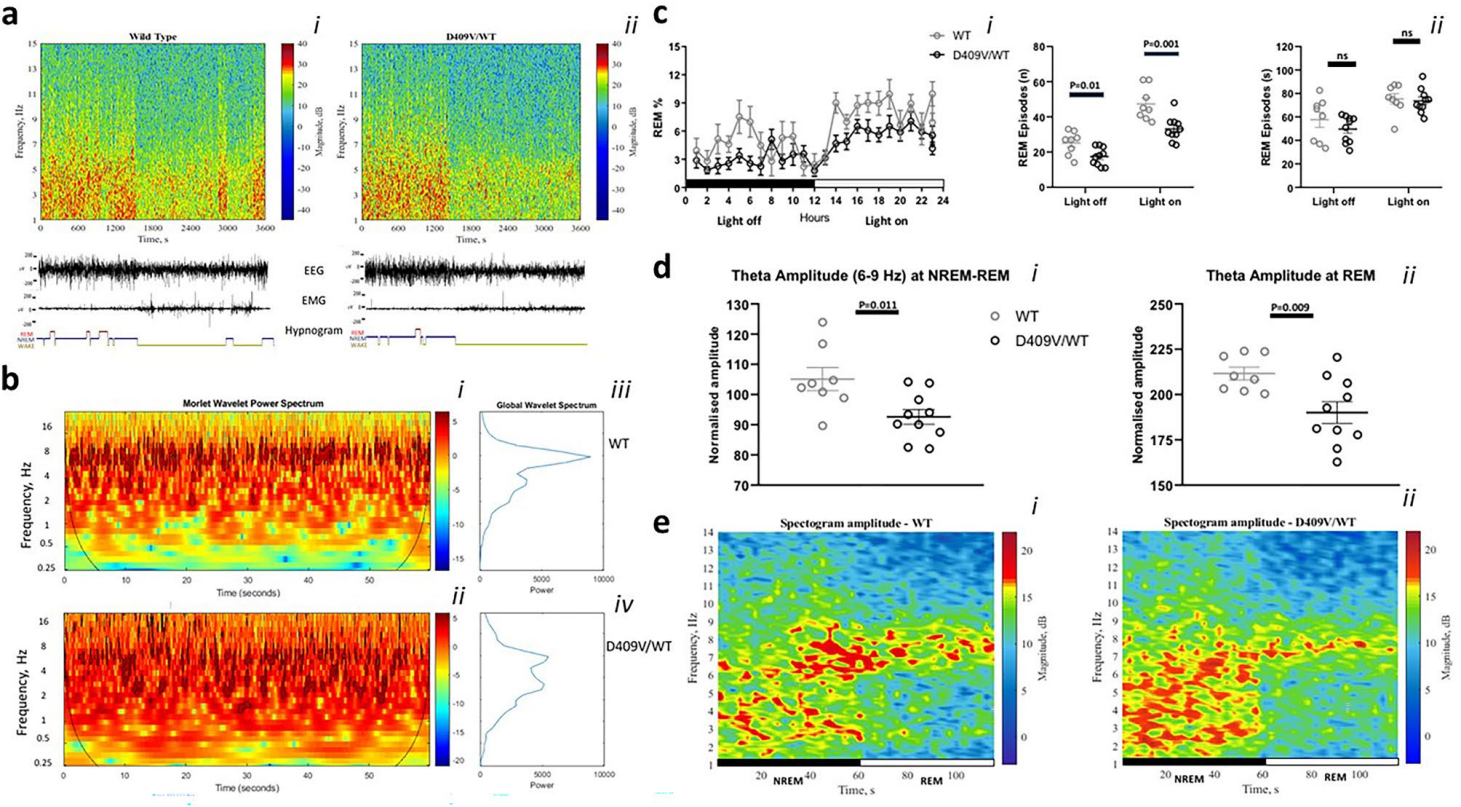
Specificity of Rapid Eye Movement (REM) sleep in D409V/WT. In (**a**), the representative EEG power, EEG/EMG traces, and hypnograms over one hour period are shown. Examples of amplitude spectograms (*right*, scale bar; dB), EEG/EMG traces, and hypnograms over one hour period at “light-on” are shown from a representative WT and D409V/WT-mouse. In (**b**), Morlet-and-Global-Wavelet-Spectrums of REM sleep in WT and D409V/WT-mice are depicted. Morlet spectrums (*right*, scale-bar; log10 relative power) were generated by averaging all REM-periods lasting one minute at “lights-on”-period; shown as a function of frequency and time (b*i, bii*). A significantly reduced power at the theta-frequency-range and an intrusion of a lower-frequency-range (1–4 Hz, delta) in D409V/WT is shown(b*i, bii*). Global-wavelet-spectrums similarly depict changes (b*iii, biv*). In (**c**), a significant reduction in REM in D409V/WT, by comparison to WT, during 24-h-EEG-recordings are shown (c*i*; *P* = .035). The decrease was due to a reduction in the number (*P* = .001 and *P* = .01; c*ii*) and not duration of REM episodes (c*iii*). In (**d**), significant reductions in theta range are shown at both NREM-to-REM-transitions (60 s; *P* = .011; d*i*) and during the subsequent REM (60–120 s; *P* = .009; d*ii*). In (**e**), similar changes by comparison to WT are depicted via representative spectograms of EEG-data-power (dB); shown as a function of frequency and time (e*i*, e*ii*).

*Firstly, in order to explore putative similarities of macroscopic and microscopic sleep structure in* D409V/WT mice^18^ *to those previously reported in iRBD*^9–11^, 24-h video-EEG recordings of aged-matched D409V/WT and wild type (WT) mice were undertaken. Increased (sleep) latency to NREM, longer and deeper bouts of NREM, overall reduced amount of REM sleep, accompanied by a distinct change in REM’s theta power and range, were all found in D409V/WT by comparison to WT mice (Fig. 1a–e).

Specifically, the time spent in NREM throughout the 24-h recording was similar between the two groups (Supplement Fig. S3). D409V/WT mice displayed a longer latency to NREM during the twelve hours lights-on (the “sleep” period) (361.6 ± 24.76 vs 505. ± 52.19 s; *P* = 0.035, t = 2.29, DF = 16; *t*-test) (Fig. S3b). However, once in NREM, D409V/WT mice remained in this state longer, as shown by longer duration of the average NREM episodes during the “sleep” period (296.5 ± 20.77 vs 357.2 ± 16.35 s; *P* = 0.033, t = 2.33, DF = 16; *t*-test) (Fig. S3c). Accordingly, sleep fragmentation and the overall number of arousals were similarly reduced (172.8 ± 10.67 vs 137.4 ± 3.53; *P* = 0.0024, t = 3.56, DF = 17; *t*-test) (Fig. S3d,e).

Strikingly, D409V/WT mice also spent significantly less time in REM (*P*(genotype)_RM-two-wayANOVA_ = 0.035; F(genotype)_1,9_ = 6.098; Fig. 1c), predominantly due to a reduction in the number of REM episodes (lights-on: 47.38 ± 3.32 vs 33.10 ± 2.19; t = 3.71, *P* = 0.001; lights-off: 25.13 ± 2.34 vs 17.40 ± 1.61, *P* = 0.0012, t = 2.79; DF = 16; *t*-test), rather than their duration (Fig. 1c).

Further spectral EEG analyses indicated a significant reduction in the theta frequency (6–9 Hz) both at the NREM-REM transitions (105.1 ± 3.76 vs 92.61 ± 2.45, *P* = 0.011, t = 2.87, DF = 16; *t*-test) and during the subsequent REM (211.6 ± 3.52 vs 190.1 ± 5.91, *P* = 0.009, t = 2.92; DF = 16; *t*-test) (Fig. 1b,d,e). Increased tonic and phasic EMG activity during REM was also observed in D409V/WT mice by comparison to WT (Supplement Fig. S4).

**Figure 2:**
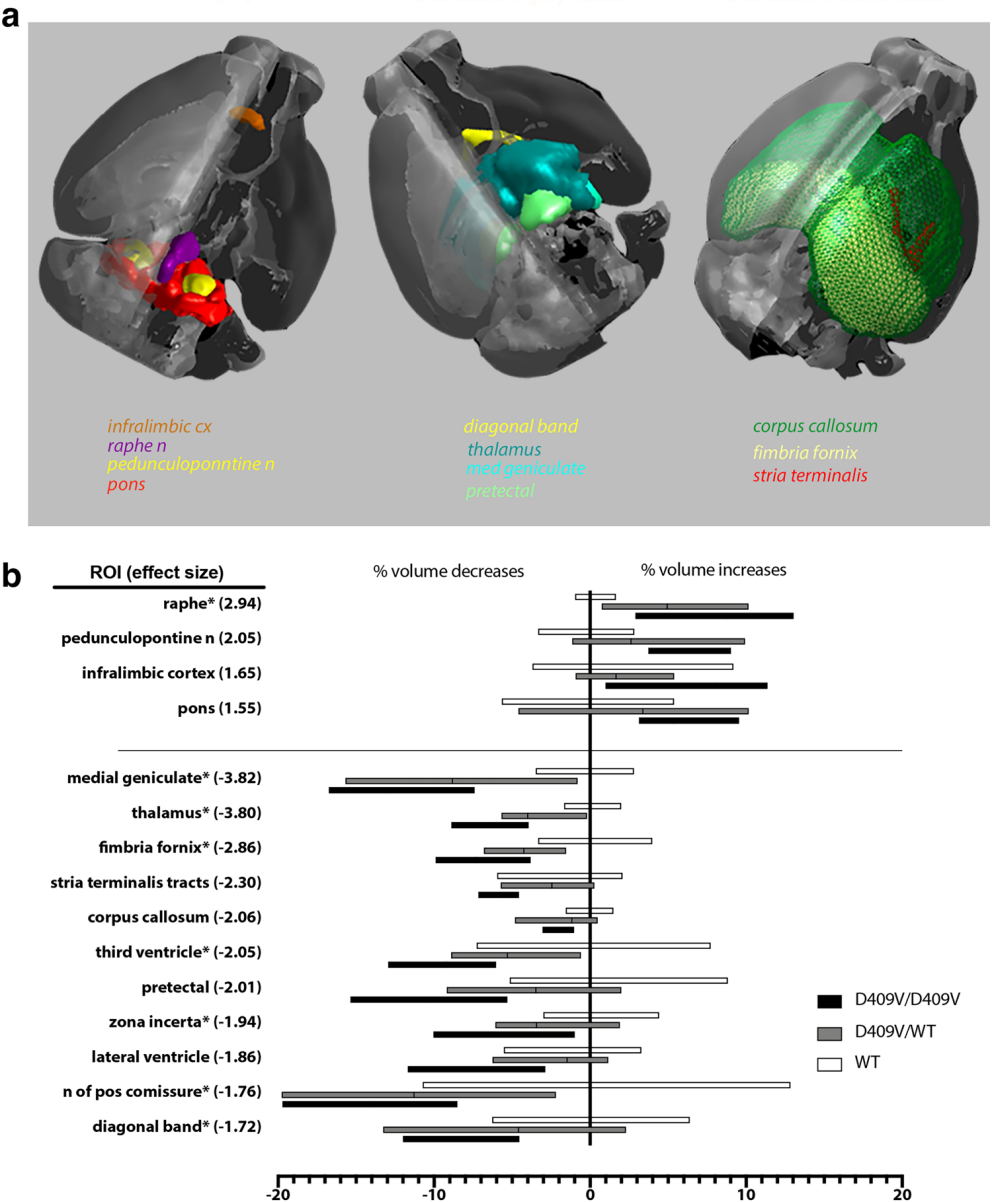
GBA1 Genotype-Driven Neuroanatomical Phenotypes. ROIs (**a**,**b**) with significant differences (*P* < .05, FDR corrected) in volume, expressed as % of the averaged wildtype group. Note relative volumes decrease with genotype (WT > D409/WT > D409/D409), except in the dorsal raphe nucleus, pedunculopontine nuclei and infralimbic cortices where there is an opposite trend (WT < D409/WT < D409/D409).

Secondly, we set to investigate how GBA genotype might affect the (sleep) neurocircuitry by conducting a high resolution ex-vivo magnetic resonance imaging (MRI) of D409V/WT, homozygous (D409V/D409V) and WT mice (Figs. 2, S5*–*S8).

Structural comparisons have highlighted GBA-genotype-driven hypotrophic changes in widespread cortical, subcortical and white matter regions (Fig. 2). Comparative enlargements were, however, also noted. For example, hypertrophic plastic changes were conspicuous in the infralimbic cortices, the pons, pedunculopontine (PPT) and the dorsal raphe nuclei (DRN; Figs. 2, S5–S8). Of note, preliminary immunochemistry findings suggest that hypertrophic changes in the PPT, albeit not in DRN, could be, at least in part, driven by the glial proliferation (Supplement Fig. S9a; *Iba1* (mean ± S.D.): WT: 138.6 ± 8.29; D409V/WT: 168.3 ± 17.78: D409V/D409V: 181 ± 36.050; *P*_D409V/WTvsWT_ = 0.014 and *P*_D409V/D409VvsWT_ = 0.021, Mann–Whitney *U*). Conversely, some of the recorded hypotrophic changes were associated with the plastic changes in the brainstem’s catecholaminergic neurocircuitry (Supplement, Fig. S9b–d). More specifically, in the dopaminergic region of the ventral tegmental area (VTA) and substantia nigra pars compacta (SNpc), a reduction in tyrosine hydroxylase-positive (TH +) area was recorded (D409V/WT:13.332 ± 2.708%; D409V/D409V:12.538 ± 1.171%; WT:14.666 ± 1.017%; *P*_D409V/D409VvsWT_ = 0.027, Mann–Whitney *U*).

Overall, the observed changes in D409V/WT mice followed the same trend towards the volume loss or gain in all regions of interest (ROIs) as recorded for D409V/D409V (Fig. 2b), suggesting a ‘dose–effect’ of the GBA-mutation on putative neurocircuitry phenotypes. However, D409V/WT-specific structural changes were the most prominent in the regions of medial geniculate nuclei, thalamus and RN, with further distinct cellular changes localised to the noradrenergic region of subcoeruleus/sublaterodorsalis nuclei A6sc (*P*_D409V/WTvsWT_ = 0.046, Mann–Whitney *U*; Supplement Fig. S9c).

## Discussion

In summary, we demonstrate here for the first time the EEG phenotype^9,10^ of GBA-mutation (D409V/WT) mouse carriers, accompanied by specific structural changes in the more widespread sleep neurocircuitry (Fig. 2). Notably, our findings are in keeping with human data for patients with GBA-mutation, where increased grey matter changes in the brain regions of DRN, midbrain, midbrain reticular formation and PPT have been recently reported^13^.

Of particular note to our study are also findings of two very recent studies^12,28^. The preliminary results of the first study, conducted in RBD patients, successfully argue for microstates of REM (e.g. phasic *versus* tonic REM) as a so far ignored, and yet likely highly informative and relatively widely accessible, EEG biomarker of RBD^12^. In a second study, Ramirez-Villegas and colleagues^28^ elegantly argue for brainstem nuclei (e.g. parabrachial and SLD; Supplement Fig. S9) as pivotal generators of the two types of ponto-geniculate-occipital (PGO) waves that subsequently direct widespread cortical NREM-REM sleep state changes^28^. It is here also suggested that putative mechanisms for such selective widespread neuronal modulations might be twofold, via cholinergic neuromodulation associated with the ascending brainstem–hippocampal synchronizing pathway that terminates in the medial septum and diagonal band of Broca, both shown as affected in our mice (Fig. 1), and secondly via direct pontine input to the hippocampus^28^. In light of these two studies, our observation of a reduction in REM-derived theta, with similar qualitative change in NREM-REM band transients in D409V/WT mice (Fig. 1), could be taken to reflect underlying changes in two types of PGO waves^28^. Moreover, this possibly also suggests an already defective pontine coordination mechanism in D409/WT mice, with likely aberrant systems and synaptic memory consolidation, as well as synaptic homeostasis. This notion is arguably also supported by our distinct neuroanatomical and histological findings that are further suggestive of an already altered function of cholinergic and noradrenergic brain networks (Fig. 1; Supplement).

In past studies of iRBD, lower number of awakenings, lower percentage of wakefulness after sleep onset, higher sleep efficiency and a higher number of sleep stage shifts have all been demonstrated in iRBD patients, which is in broad keeping with our EEG findings for the D409/WT model (Figs. 1, S3)^9,11^. However, in contrast to our findings are results from human studies that overwhelmingly report a relatively preserved sleep architecture in iRBD patients^9,29^, albeit with varied degrees of age-dependant EEG slowing during wake and REM sleep^10,30,31^. Notwithstanding, contradictory and varied human sleep EEG findings have, however, been reported over the years^9,10^. For example, in opposition, other studies have reported an altered density of NREM sleep spindles^32^ and an increased percentage of slow-wave sleep in patients with iRBD^33,34^. Similarly, others have demonstrated an increased all-night δ power in iRBD^9,35^. Correspondingly, a reduced decline in SWA from early to late NREM sleep, along with reduced change in the distribution of the amplitude of slow waves have recently been reported^12^. Nonetheless, to date, the majority of studies appear to report only minimal or absent abnormality of EEG during NREM^36^.

In regards to REM sleep, several earlier studies have demonstrated substantial EEG abnormality during REM in iRBD^10^. For instance, impaired cortical activation during both wakefulness and REM in iRBD has been shown, including progressive NREM, REM and transitions instability; although changes in sleep macroarchitecture have been far less consistently demonstrated^9,10,30,31^. Thus, despite some intriguing similarities, the EEG findings of D409V/WT mice also differ to those previously reported in iRBD. Arguably, this may, at least in part, reflect the distinct sleep pathology of D409V/WT model.

Therefore our findings may, conceivably, represent some of the earliest pathologic EEG changes that occur in distinct relation to GBA1 mutation. As such, they may essentially diverge from those that occur in α-synucleinopathies of other aetiologies. Thus far, very little is understood about the relationship between α-synuclein and GBA, although a strong link has been established between its deficiency and the deposition of aggregated α-synuclein^37^. However, it is far from clear whether this relationship is consequence of a loss, or gain of, function of GBA due to heterozygous GBA1 mutation^37,38^, and even less is known of its impact on the sleep physiology.

Finally, the loss of REM sleep atonia control is well accepted in iRBD patients^10^, although the underlying mechanism remains ambiguous^3^. Previous studies have linked a dysfunction of pontine sublateral dorsal/subcoeruleus nucleus, also shown as affected in our study (Supplement, Fig. S9), with loss of REM atonia^3,39^. According to the current human sleep scoring regulations, in order to distinguish RBD patients reliably, (tonic/phasic) chin EMG activity should be scored in 30-s epochs combined with bilateral phasic activity of the flexor digitorum superficialis muscles in more than 27% of REM sleep^3,10^. As yet, no such scoring guidance exists for animal (rodent) RBD model. In our study, though, we observed an intermittent excessive fragmentary myoclonus in nuchal muscles of D409V/WT mice (Fig. S4), presumably in keeping to that reported to occur in prodromal iRBD^3^. Nonetheless, we find that due to the technical limitations of our study any causal extrapolation is herein limited. For instance, we did not map or analyse the (age-related) phasic and tonic involvement of various rodent muscle groups. This would be important to do in future studies in order to obtain the most optimal EMG biomarker that meaningfully reflects presumed neurodegenerative changes.

Despite obvious limitations of our preliminary and cross-sectional study, we believe that the findings posit important translational questions in regards to whether the reported changes in REM-sleep and its theta-frequency-range power^20^, along with involvement of core circuitry structures such are PPT, RN and SLD suggest a differential phenotype of any early non-motor (e.g. cognitive, memory and mood) symptoms in non-manifesting human GBA carriers. Similarly, we believe that these findings also advance the relevance of the GBA1 mice as a valuable model for future exploration of neurostructural and physiological targets in iRBD, and its links with other α-synucleinopathies. Future studies should aim to delineate authoritative age-related EEG changes in this animal model, ideally guided and informed by the concomitant multidisciplinary and multi-centre translational trials of RBD patients with different GBA variants.

## Supplementary Information

Supplementary Information.

## Data Availability

All data that support the findings of this study are available upon reasonable request from the corresponding author.

## References

[1] Haba-Rubio J (2018). Prevalence and determinants of rapid eye movement sleep behavior disorder in the general population. Sleep.

[2] Sasai-Sakuma T, Takeuchi N, Asai Y, Inoue Y, Inoue Y (2020). Prevalence and clinical characteristics of REM sleep behavior disorder in Japanese elderly people. Sleep.

[3] Hogl B, Stefani A, Videnovic A (2018). Idiopathic REM sleep behaviour disorder and neurodegeneration—An update. Nat Rev Neurol.

[4] Postuma RB (2014). Prodromal Parkinson’s disease—Using REM sleep behavior disorder as a window. Parkinsonism Relat. Disord..

[5] Galbiati A, Verga L, Giora E, Zucconi M, Ferini-Strambi L (2019). The risk of neurodegeneration in REM sleep behavior disorder: A systematic review and meta-analysis of longitudinal studies. Sleep Med. Rev..

[6] Krohn L (2020). GBA variants in REM sleep behavior disorder: A multicenter study. Neurology.

[7] Gan-Or Z (2015). GBA mutations are associated with rapid eye movement sleep behavior disorder. Ann. Clin. Transl. Neurol..

[8] Wasserman D (2022). Restricted truncal sagittal movements of rapid eye movement behaviour disorder. npj Parkinson’s Dis..

[9] Ferri R (2017). REM sleep EEG instability in REM sleep behavior disorder and clonazepam effects. Sleep.

[10] Inoue Y, Sasai T, Hirata K (2015). Electroencephalographic finding in idiopathic REM sleep behavior disorder. Neuropsychobiology.

[11] Christensen JAE (2016). Sleep stability and transitions in patients with idiopathic REM sleep behavior disorder and patients with Parkinson’s disease. Clin. Neurophysiol..

[12] Valomon A (2021). A high-density electroencephalography study reveals abnormal sleep homeostasis in patients with rapid eye movement sleep behavior disorder. Sci. Rep..

[13] Yousaf, T., Kershaw, M., Suarez Contreras, V., Vickers, P., Pagano, G. & Politis, M. Structural changes in non-manifesting GBA mutation carriers and GBA mutation carriers with Parkinson’s disease. *Mov. Disord.* **34**. https://movementdisorders.onlinelibrary.wiley.com/doi/10.1002/mds.27795 (2019).

[14] Baker KG, Tork I, Hornung JP, Halasz P (1989). The human locus coeruleus complex: An immunohistochemical and three dimensional reconstruction study. Exp. Brain Res..

[15] Peever J, Luppi PH, Montplaisir J (2014). Breakdown in REM sleep circuitry underlies REM sleep behavior disorder. Trends Neurosci..

[16] Ramaligam V, Chen MC, Saper CB, Lu J (2013). Perspectives on the rapid eye movement sleep switch in rapid eye movement sleep behavior disorder. Sleep Med..

[17] Ehrminger M (2016). The coeruleus/subcoeruleus complex in idiopathic rapid eye movement sleep behaviour disorder. Brain.

[18] Clarke E (2019). Age-related neurochemical and behavioural changes in D409V/WT GBA1 mouse: Relevance to lewy body dementia. Neurochem. Int..

[19] Sardi SP (2011). CNS expression of glucocerebrosidase corrects alpha-synuclein pathology and memory in a mouse model of Gaucher-related synucleinopathy. Proc. Natl. Acad. Sci. U. S. A..

[20] Kim B (2017). Differential modulation of global and local neural oscillations in REM sleep by homeostatic sleep regulation. Proc. Natl. Acad. Sci. U. S. A..

[21] Van Gelder RN, Edgar DM, Dement WC (1991). Real-time automated sleep scoring: Validation of a microcomputer-based system for mice. Sleep.

[22] Torrence C, Compo GP (1997). A practical guide to wavelet analysis. Bull. Am. Meteorol. Soc..

[23] Zhivomirov H (2019). On the development of STFT-analysis and ISTFT-synthesis routines and their practical implementation. TEM Journal.

[24] Gelegen C (2018). Excitatory pathways from the lateral habenula enable propofol-induced sedation. Curr Biol.

[25] Gelegen C (2014). Staying awake—A genetic region that hinders alpha2 adrenergic receptor agonist-induced sleep. Eur. J. Neurosci..

[26] Wood TC (2016). Whole-brain ex-vivo quantitative MRI of the cuprizone mouse model. PeerJ.

[27] Kilkenny C, Browne WJ, Cuthill IC, Emerson M, Altman DG (2010). Improving bioscience research reporting: The ARRIVE guidelines for reporting animal research. PLoS Biol.

[28] Ramirez-Villegas JF, Besserve M, Murayama Y, Evrard HC, Oeltermann A, Logothetis NK (2021). Coupling of hippocampal theta and ripples with pontogeniculooccipital waves. Nature.

[29] Wasserman D (2021). Striatal dopaminergic deficit and sleep in idiopathic rapid eye movement behaviour disorder: An explorative study. Nat. Sci. Sleep.

[30] Sasai T, Matsuura M, Inoue Y (2013). Electroencephalographic findings related with mild cognitive impairment in idiopathic rapid eye movement sleep behavior disorder. Sleep.

[31] Iranzo A (2010). Electroencephalographic slowing heralds mild cognitive impairment in idiopathic REM sleep behavior disorder. Sleep Med..

[32] O'Reilly C, Godin I, Montplaisir J, Nielsen T (2015). REM sleep behaviour disorder is associated with lower fast and higher slow sleep spindle densities. J. Sleep Res..

[33] Oksenberg A, Radwan H, Arons E, Hoffenbach D, Behroozi B (2002). Rapid Eye Movement (REM) sleep behavior disorder: A sleep disturbance affecting mainly older men. Isr. J. Psychiatry Relat. Sci..

[34] Olson EJ, Boeve BF, Silber MH (2000). Rapid eye movement sleep behaviour disorder: Demographic, clinical and laboratory findings in 93 cases. Brain.

[35] Massicotte-Marquez J (2005). Slow-wave sleep and delta power in rapid eye movement sleep behavior disorder. Ann. Neurol..

[36] Latreille V, Carrier J, Montplaisir J, Lafortune M, Gagnon JF (2011). Non-rapid eye movement sleep characteristics in idiopathic REM sleep behavior disorder. J. Neurol. Sci..

[37] Stojkovska I, Krainc D, Mazzulli JR (2018). Molecular mechanisms of alpha-synuclein and GBA1 in Parkinson’s disease. Cell Tissue Res..

[38] Clarke, E. The unfolded protein response: A potential link between heterozygous mutations in GBA1 and Lewy body dementia? In *Wolfson Centre for Age Related Diseases* (King’s College London, 2018).

[39] Chan PC, Lee HH, Hong CT, Hu CJ, Wu D (2018). REM sleep behavior disorder (RBD) in dementia with Lewy bodies (DLB). Behav. Neurol..

